# Honokiol ameliorates angiotensin II‐induced hypertension and endothelial dysfunction by inhibiting HDAC6‐mediated cystathionine γ‐lyase degradation

**DOI:** 10.1111/jcmm.15686

**Published:** 2020-08-04

**Authors:** Zhexi Chi, Truc Phan Hoang Le, Sang Ki Lee, Erling Guo, Dongsoo Kim, Sanha Lee, Seung‐Yong Seo, Sook Young Lee, Jae Hyung Kim, Sang Yoon Lee

**Affiliations:** ^1^ Department of Anesthesiology and Pain Medicine Ajou University School of Medicine Suwon Korea; ^2^ Department of Biomedical Sciences Ajou University Graduate School of Medicine Suwon Korea; ^3^ Department of Sport Science Chungnam National University Daejeon Korea; ^4^ Department of Anesthesiology and Pain Medicine Hallym University Dongtan Sacred Heart Hospital Hwaseong Korea; ^5^ College of Pharmacy Gachon University Incheon Korea; ^6^ Institute for Medical Sciences Ajou University School of Medicine Suwon Korea

**Keywords:** acetylation, angiotensin II, cystathionine γ‐lyase, histone deacetylase 6, honokiol, hydrogen sulphide, hypertension

## Abstract

Hypertension and endothelial dysfunction are associated with various cardiovascular diseases. Hydrogen sulphide (H_2_S) produced by cystathionine γ‐lyase (CSE) promotes vascular relaxation and lowers hypertension. Honokiol (HNK), a natural compound in the *Magnolia* plant, has been shown to retain multifunctional properties such as anti‐oxidative and anti‐inflammatory activities. However, a potential role of HNK in regulating CSE and hypertension remains largely unknown. Here, we aimed to demonstrate that HNK co‐treatment attenuated the vasoconstriction, hypertension and H_2_S reduction caused by angiotensin II (AngII), a well‐established inducer of hypertension. We previously found that histone deacetylase 6 (HDAC6) mediates AngII‐induced deacetylation of CSE, which facilitates its ubiquitination and proteasomal degradation. Our current results indicated that HNK increased endothelial CSE protein levels by enhancing its stability in a sirtuin‐3‐independent manner. Notably, HNK could increase CSE acetylation levels by inhibiting HDAC6 catalytic activity, thereby blocking the AngII‐induced degradative ubiquitination of CSE. CSE acetylation and ubiquitination occurred mainly on the lysine 73 (K73) residue. Conversely, its mutant (K73R) was resistant to both acetylation and ubiquitination, exhibiting higher protein stability than that of wild‐type CSE. Collectively, our findings suggested that HNK treatment protects CSE against HDAC6‐mediated degradation and may constitute an alternative for preventing endothelial dysfunction and hypertensive disorders.

## 1 INTRODUCTION

Hypertension (high blood pressure) confers high risk for various types of diseases afflicting elderly individuals with chronic hypertension, representing a leading cause of cardiovascular disorders including stroke and heart failure.^1^, ^2^ In turn, the dilation and constriction of arterial blood vessels serves as a critical factor regulating blood pressure.^3^ Notably, the gaseous transmitter hydrogen sulphide (H_2_S) is recognized as an endogenous, physiological vasodilator that contributes to lower blood pressure.^3^, ^4^ Moreover, H_2_S exhibits anti‐inflammatory and anti‐oxidative activities and mediates protein S‐sulphydration.^4^, ^5^, ^6^

Cystathionine γ‐lyase (CSE), encoded by the *CTH* gene, is a major H_2_S‐producing enzymes.^7^ CSE (also termed γ‐cystathionase) and cystathionine β‐synthase, which together constitute the transsulphuration pathway, mediate successive metabolic conversions of homocysteine and cysteine via the intermediate product cystathionine.^8^ Mutations in *CTH* cause the γ‐cystathionase deficiency syndrome cystathioninuria, an autosomal recessive genetic disorder, whereas CSE deletion results in hypertension and atherosclerosis with endothelial dysfunction.^7^, ^9^, ^10^ Alternatively, numerous studies have demonstrated that CSE‐mediated H_2_S generation induces endothelium‐dependent vasodilation and improves cardiovascular function and integrity.^11^, ^12^

Histone deacetylase 6 (HDAC6) is implicated in the pathophysiology of hypertension‐related vascular diseases.^13^, ^14^ HDAC6 plays a significant role in the cardiac dysfunction mediated by angiotensin II (AngII), an inducer of hypertension.^15^, ^16^ An increased HDAC6 expression mediated by the atherogenic factor oxidized low‐density lipoprotein impaired CSE function and vasorelaxation.^17^ In similar contexts, HDAC6 has been proposed as a therapeutic target for treatment of cardiovascular diseases.^15^, ^16^, ^17^, ^18^, ^19^ We also recently reported that tubastatin A, an HDAC6‐specific inhibitor, could increase CSE acetylation and enhance its protein levels and H_2_S production, thereby helping to attenuate the vasoconstriction and hypertension induced by AngII.^20^ Moreover, HDAC6, a member of the class IIb HDAC family, exhibits distinct characters compared to other HDAC family members in that it has unique specificity for protein deacetylation of non‐histone substrates such as α‐tubulin, cortactin and heat‐shock protein 90.^21^, ^22^, ^23^

Honokiol (HNK) is a natural product isolated from the phenolic extracts of the plant *Magnolia officinalis*. Accumulating evidence indicates that HNK potently suppresses oxidative and inflammatory responses.^24^, ^25^ Such beneficial, therapeutic capacities of HNK are mediated by its broad functionalities to modulate a wide range of signalling proteins. HNK also serves as a phytochemical to prevent cardiovascular diseases such as cardiac hypertrophy by activating mitochondrial sirtuin‐3 (SIRT3), a member of the sirtuin family that belongs to class III HDACs.^24^, ^26^, ^27^

It appears that both H_2_S and HNK retain cardioprotective activities. Currently, however, few studies have evaluated HNK effects on CSE‐mediated H_2_S production. Moreover, relatively little is known about the regulatory role of HNK in AngII‐induced hypertension. In this study, we examined the potential effects of HNK on hypertension, vascular function and H_2_S production using mice and aortic endothelial cells treated with AngII. We also investigated the molecular details regarding the role of HNK in regulating HDAC6 activity and CSE protein stability, as these are associated with CSE acetylation and ubiquitination.

## 2 MATERIALS AND METHODS

### 2.1 Chemicals

Most chemicals including AngII, cycloheximide (CHX), MG132, acetylcholine (ACh), sodium nitroprusside (SNP) and Dulbecco's modified Eagle's medium were obtained from Sigma‐Aldrich (St. Louis, MO, USA). Lipofectamine 2000, Lipofectamine RNAiMAX and Opti‐MEM I were obtained from Thermo Fisher Scientific (Waltham, MA, USA). HNK [2‐(4‐hydroxy‐3‐prop‐2‐enyl‐phenyl)‐4‐prop‐2‐enyl‐phenol] (#S2310) and tubastatin A (#S8049) were purchased from Selleckchem (Houston, TX, USA) and dissolved in dimethyl sulphoxide (DMSO).

### 2.2 AngII‐infused hypertensive mice and HNK injection

C57BL/6N male mice (twelve‐week‐old; Orient Bio, Seongnam, Republic of Korea) were handled according to the guidelines of the Institutional Animal Care and Use Committee and the experimental procedures were approved by the Laboratory Animal Research Center of Ajou University Medical Center (approval ID: 2016‐0065). After anaesthesia with isoflurane solution, mice were infused with AngII (1 mg/kg^/^day) for 4 weeks through micro‐osmotic pumps (Alzet model 1004; Cupertino, CA, USA) subcutaneously implanted into the back of the mice. As controls, mice were subjected to the same surgical operation without pump insertion. HNK (2 mg/kg^/^day) or phosphate‐buffered saline was intraperitoneally injected into the AngII‐treated mice once a day.

### 2.3 Blood pressure measurement

We measured blood pressure in the hypertensive mice before and 2 or 4 weeks after AngII osmotic pump insertion via a non‐invasive CODA tail‐cuff blood pressure monitor system (Kent Scientific, Torrington, CT, USA).^20^ The volume pressure recording cuff was placed on the tail and changes in systolic, diastolic and mean blood pressure were measured on a heated platform set at 37°C. Mice were trained in a holder every day for 2 weeks and maintained in a quiet and dark location. The average of ten readings was used for the analysis.

### 2.4 Force tension myography

We performed myographic measurements according to previously described procedures.^17^, ^20^ Briefly, mice had been treated with AngII and/or HNK for 4 weeks as described in Section 2.2 and aorta samples were then isolated. The isometric contractions were measured via a wire myograph system (model 620 DMT, Danish Myo Technology, Aarhus, Denmark). Aortic rings were challenged with 60 mmol/L KCl and the passive stretch was repeated after a wash with Krebs buffer, followed by equilibrium for 60 minutes. The vessels were pre‐constricted with phenylephrine (1 μmol/L) for 15 minutes. We then monitored dose‐dependent (0.1 nmol/L–10 μmol/L) responses to ACh followed by SNP after the washes and re‐equilibrium.

### 2.5 Cell cultures and treatments

Human aortic endothelial cells (HAECs) and human embryonic kidney 293 (HEK293) cells were cultured as described previously.^20^ Cells were routinely subcultured at 3‐day intervals and three to five passages of cell cultures were used in all experiments. For sample preparation, equal numbers of cells were plated into culture dishes at a density of 5 × 10^4^ cells/cm^2^ overnight and then treated with AngII (100 nmol/L), HNK (5 μmol/L) and/or other chemicals as indicated. For AngII and HNK co‐treatment, cells were pretreated with HNK for 1 hour before AngII treatment for 12 hour.

### 2.6 H_2_S measurement

H_2_S levels in HAECs treated with AngII and/or HNK were determined using the agar trapping method as previously described.^20^, ^28^ Briefly, H_2_S trapped as zinc sulphide was converted into methylene blue through chemical reactions. In the case of aorta samples, mouse aortas were isolated from control mice before treatments with AngII and/or HNK.

### 2.7 Antibodies and plasmids

Antibodies against HDAC6 (#7558), acetylated lysine (Ace‐K, #9441), SIRT3 (#2627), endothelial nitric oxide synthase (eNOS, #32027), phospho‐eNOS (Ser1177, #9571) and acetyl‐α‐tubulin (Lys40, #12152) were obtained from Cell Signaling Technology (Danvers, MA, USA). Myc (#sc‐40), β‐actin (#sc‐1616) and CSE (#sc‐374249) antibodies were purchased from Santa Cruz Biotechnology (Dallas, TX, USA). Antibodies to HA (#MMS‐101R, Covance, Princeton, NJ, USA), FLAG (#F1804, Sigma‐Aldrich), α‐tubulin (#T5168, Sigma‐Aldrich) and CSE (#12217‐1‐AP, ProteinTech, Rosemont, IL, USA) were commercially purchased. Expression plasmids pcDNA‐HDAC6‐FLAG (#30482), pcDNA‐HDAC6.DC‐FLAG (#30483), pcDNA3.1‐SIRT3‐FLAG (#13814) and pRK5‐HA‐ubiquitin (#17608) were obtained from Addgene (Cambridge, MA, USA). The CSE‐Myc plasmid (#RC231191) was purchased from OriGene Technologies (Rockville, MD, USA). The K73R mutation in the CSE‐Myc template was generated using forward (5′‐GGAATTGCCTTGAAAGAGCAGTGGCAGCACTGG‐3′) and reverse (5′‐CCAGTGCTGCCACTGCTCTTTCAAGGCAATTCC‐3′) primers from Bioneer (Daejeon, Republic of Korea) and the QuikChange II Site‐Directed Mutagenesis Kit (Agilent Technologies, Santa Clara, CA, USA) according to manufacturer protocol. All plasmids were purified using an EndoFree Plasmid Maxi Kit (Qiagen, Valencia, CA, USA).

### 2.8 Transfection and gene knockdown

HDAC6, CSE and/or ubiquitin expression plasmids or corresponding empty vectors were transfected into HEK293 cells or HAECs using Lipofectamine 2000 for 1 day. For gene knockdown, double‐stranded small interfering RNA (siRNA) targeting *CSE* (5′‐GGUUAUUUAUCCUGGGCUGUU‐3′) or *SIRT3* (5′‐CUGUGCCUAGUUGAACGGCAA‐3′), or control siRNA (5′‐UUCUCCGAACGUGUCACGUUU‐3′) from Bioneer were mixed with Lipofectamine RNAiMAX in Opti‐MEM I and the mixtures were added to cells for 2 days.

### 2.9 Western blotting and immunoprecipitation (IP)

Cell lysate preparation, protein quantification, sodium dodecyl sulphate‐polyacrylamide gel electrophoresis, Western blotting and IP were performed as described previously.^29^ The lysis buffer was freshly supplemented with *N*‐ethylmaleimide (10 mmol/L) for detecting CSE ubiquitination. For IP of endogenous CSE and transfected CSE‐Myc, cell lysates (1.5 mg) were incubated with 5 μg anti‐CSE and anti‐Myc mouse monoclonal antibody, respectively (4 hours, 4°C). The immune complexes were captured with 30 μL Protein A/G PLUS‐Agarose IP reagent (Santa Cruz Biotechnology) for an additional 2 hours and washed 5 times with lysis buffer.

### 2.10 Quantitative real‐time PCR

Total RNA was purified using the RNeasy Mini Kit (Qiagen), and cDNA was synthesized from the total RNA (1 μg) using ReverTra Ace qPCR RT Master Mix (Toyobo, Osaka, Japan). Then, quantitative real‐time reverse transcription PCR (qRT‐PCR) was performed using an ABI7900 qPCR machine (Applied Biosystems, Foster City, CA, USA) with TOPreal qPCR 2X PreMIX SYBR Green and low ROX reagent (Enzynomics, Daejeon, Republic of Korea). The following specific primers (Cosmo Genetech, Seoul, Republic of Korea) were used: *CSE* (forward) 5′‐GGTTTCCTGCCACACTTCCA‐3′ and (reverse) 5′‐CATCCAGTGCTGCCACTGCT‐3′; and *GAPDH* (forward) 5′‐AAAATCAAGTGGGGCGATGC‐3′ and (reverse) 5′‐AGGAGGCATTGCTGATGATCT‐3′. All PCR samples were prepared in triplicate and the relative mRNA expression levels were normalized to *GAPDH* and determined using the 2^−ΔΔCt^ method.

### 2.11 **HNK**–**HDAC6 binding**

HNK binding to HDAC6 was tested using the EpiQuik HDAC6 Assay Kit (EpiGentek #P‐4046, Farmingdale, NY, USA) following manufacturer protocol with slight modification. HNK (final 10 μmol/L), rather than cell lysates, was directly added to the plate wells coated with the unique HDAC6 affinity substrate and incubated with the purified HDAC6 control protein (160 ng; 2 hours, 37°C). Alternatively, we applied a synthetic biotin‐labelled HNK.^30^ Following HAEC treatment with biotin‐labelled HNK (5 μmol/L) for 12 hours, cell lysates (0.6 mg) were mixed with 25 μL streptavidin agarose resin (#20347, Thermo Fisher Scientific) overnight at 4°C, and then, the resulting pull‐down samples were processed for immunoblot analysis.

### 2.12 HDAC6 activity assay

HDAC6 deacetylase activity was tested using a fluorometric HDAC6 Activity Assay Kit (BioVision #K466‐100, Milpitas, CA, USA) according to manufacturer protocol. Briefly, in vitro reaction mixtures (100 μL) containing the human HDAC6 enzyme and a synthetic acetylated peptide substrate were incubated without or with HNK for 30 minutes at 37°C. The fluorescent intensities were measured using a microplate reader (Synergy H1 model, BioTek Instruments, Winooski, VT, USA) at 380 nm excitation and 490 nm emission.

### 2.13 Statistical analysis

All experiments were performed independently at least three times with similar results. Band intensities of Western blots were measured using ImageJ software (National Institutes of Health, Bethesda, MD, USA). Data shown in the graphs are presented as the means ± SEM Statistical significance was determined by one‐way analysis of variance with Tukey's multiple comparison tests or the indicated statistical methods using GraphPad Prism 7 software (La Jolla, CA, USA).

## 3 RESULTS

### 3.1 HNK attenuates AngII‐induced hypertension and vascular endothelial dysfunction

**FIGURE 1:**
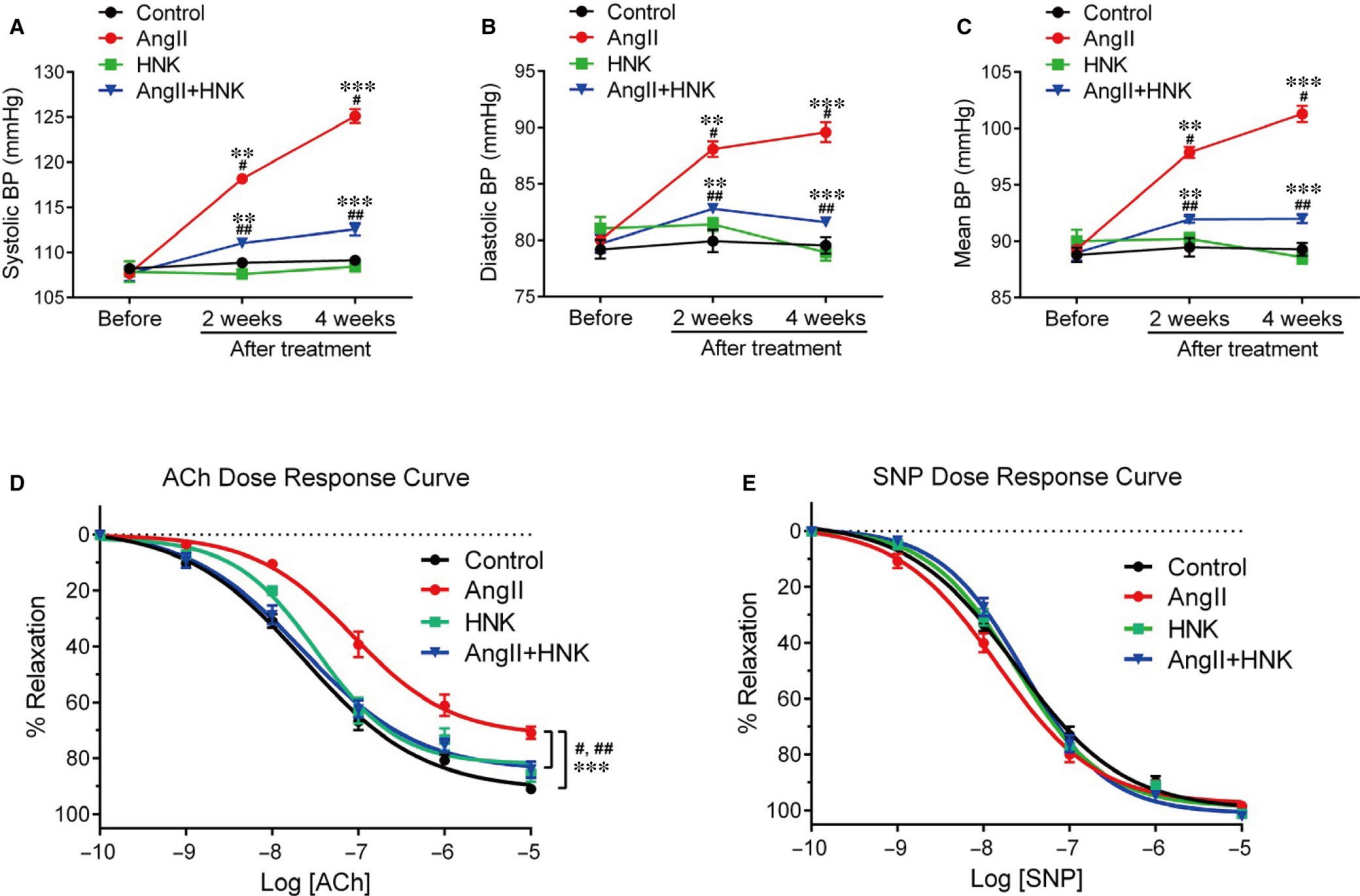
Antihypertensive and vasorelaxatory effects of HNK on AngII‐treated mice. Four groups of mice including untreated controls and mice infused with AngII via osmotic pump and/or HNK via peritoneal injection up to 4 wk were subjected to blood pressure measurements using a tail‐cuff monitoring system. Seven mice per group were used. Changes in systolic (A), diastolic (B) and mean (C) blood pressure were measured prior to and 2 or 4 wk following the delivery of AngII and/or HNK. Values represent the means ± SEM. ^#^AngII vs control, ^##^AngII vs AngII + HNK. **P < 0.01, ***P < 0.001. (D,E) Aortas were isolated from the mice treated with AngII and/or HNK in the same manner as described above and then processed for vascular reactivity measurements using wire myography. The dose‐dependent responses to ACh (D) and SNP (E) were monitored in the indicated four mouse groups. Three to four aortic rings per mouse were used. The group data were analysed by logistic dose‐response curves with four parameters using GraphPad Prism 7 software. The relaxation responses were prepared by nonlinear regression (curve fit) method and calculated as a percentage of tension after pre‐construction using 1 μmol/L phenylephrine. Statistical significance of the indicated data sets was determined using least square fit and the extra sum‐of‐squares F test. Values represent the means ± SEM. ^#^AngII vs control, ^##^AngII vs AngII + HNK. ***P < 0.001. Statistical significance was determined using the Holm‐Sidak method

AngII, a potent hypertensive agent, induces vascular constriction.^15^, ^31^ To evaluate a potential effect of HNK on hypertension, we first performed tail‐cuff measurements of blood pressure in in vivo AngII‐ and/or HNK‐infused mice. AngII and HNK were chronically administered for up to 4 weeks via osmotic pump and peritoneal injection, respectively. Systolic (Figure 1A), diastolic (Figure 1B) and mean (Figure 1C) blood pressure of mouse tails was increased at 2 and 4 weeks after AngII treatment, as expected. Conversely, AngII‐mediated blood pressure increase was blunted upon HNK co‐administration (Figure 1A–C).

We then examined whether HNK could modulate vascular reactivity by performing myographic measurements of ex vivo aorta rings that were isolated from the AngII‐ and/or HNK‐treated mice used for the blood pressure measurements. Consistent with the ability of ACh to induce vascular relaxation by stimulating endothelial nitric oxide (NO) production,^12^, ^17^ ACh (0.1 nmol/L–10 μmol/L) dose‐dependently induced the relaxation of aortic rings pre‐constricted with 1 μmol/L phenylephrine in the control group whereas AngII significantly decreased the ACh‐induced relaxation (Figure 1D). Conversely, HNK co‐administration improved the AngII‐attenuated vasorelaxation (Figure 1D). We also monitored dose‐dependent responses to the exogenous NO donor SNP to assess endothelium‐independent relaxation of aorta rings.^17^ In contrast to the results from ACh stimulation, AngII and/or HNK did not significantly affect the SNP dose responses (Figure 1E). These results supported a role for HNK in suppressing AngII‐induced endothelium‐dependent vasoconstriction. NO produced by eNOS is a critical mediator of vasorelaxation.^32^, ^33^ Thus, we examined whether HNK could affect eNOS activity by detecting changes in phospho‐eNOS (S1177), an active form of eNOS.^34^ HNK treatment induced dose‐dependent increases in phospho‐eNOS levels (Figure S1A). AngII down‐regulated the phospho‐eNOS levels (Figure S1B), which was consistent with the previous results.^35^ HNK was also shown to block the AngII‐induced decrease in phospho‐eNOS levels (Figure S1B). These suggested a contribution of HNK to eNOS activation.

### 3.2 HNK recovers H_2_S levels decreased by AngII

**FIGURE 2:**
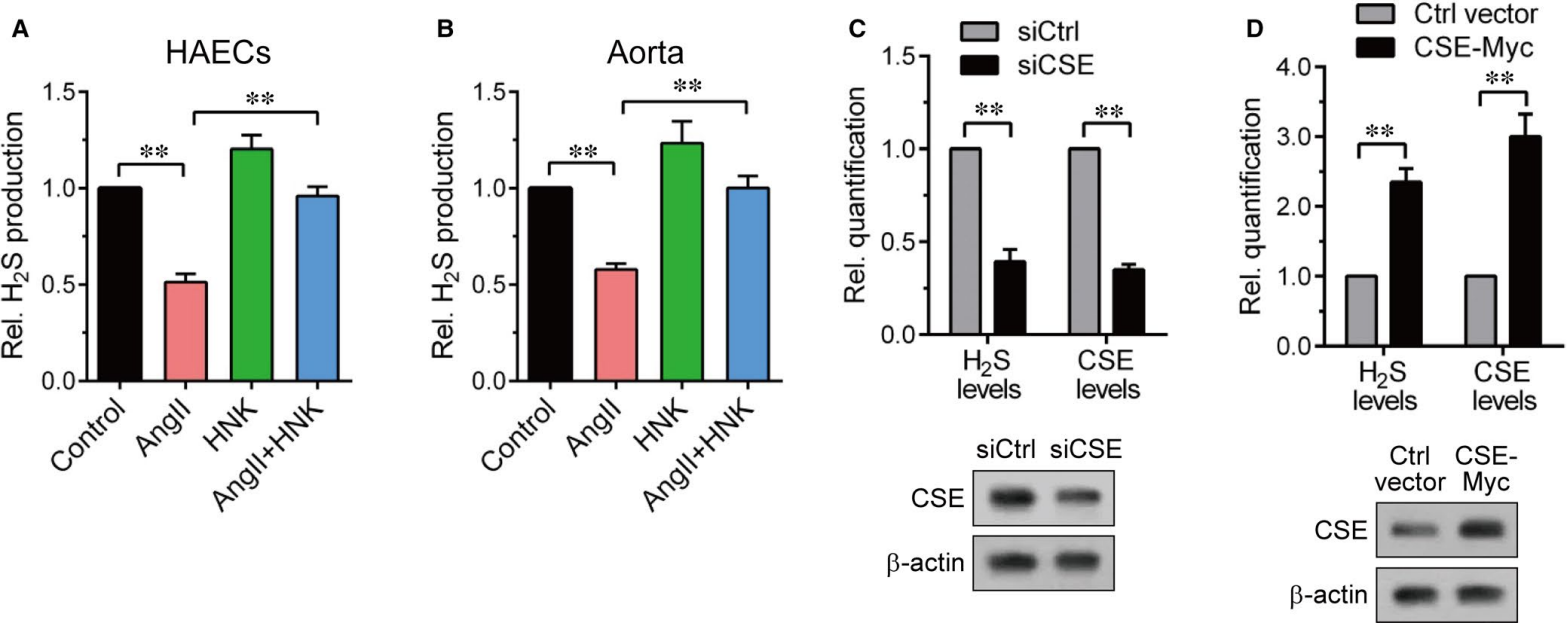
Opposing effects of HNK on AngII‐induced H_2_S reduction. A, HAEC (n = 9) or B, isolated mouse aortas (n = 6) were treated with DMSO or HNK (5 μmol/L) for 1 h and then incubated in the absence or presence of 100 nmol/L AngII for 12 h. H_2_S was collected using an agar trapping method and detected by an in situ methylene blue assay as described in Materials and Methods. H_2_S levels were calculated by using the linear range of a standard curve obtained with NaHS and quantified relative to the control. Values represent the means ± SEM. Statistical significance was determined using Dunn's multiple comparison test. **P < 0.01. C, HEK293 cells were transfected with CSE siRNA (siCSE) or control siRNA (siCtrl) for 2 d. D, HEK293 cells were transfected with CSE‐Myc or control vector plasmid for 1 d. (C,D) H_2_S levels were measured in the same manner as in (A). The resulting cell lysates were analysed using CSE and β‐actin (a loading control) immunoblotting. H_2_S levels and CSE protein levels were quantified relative to the control. Values represent the means ± SEM. **P < 0.01

H_2_S lowers blood pressure and induces vasorelaxation whereas AngII decreases H_2_S levels in HAECs and mouse aorta.^7^, ^12^, ^20^ We thus tested whether HNK could affect H_2_S production using an agar trap method employing an in situ methylene blue assay that allowed sensitive detection of H_2_S generated from cultured cells.^28^ In HAECs, the decreased H_2_S levels 12 hours after AngII treatment were recovered by 1‐h HNK pretreatment (Figure 2A). To obtain H_2_S measurement with ex vivo aorta samples, we firstly isolated murine aortas then treated them with AngII and/or HNK. We obtained similar results showing that HNK antagonized the reducing effect of AngII on H_2_S production (Figure 2B). Because endothelial H_2_S produced by CSE mediates vasodilatory effects, we performed H_2_S detection via the agar trap method in CSE knockdown or CSE‐overexpressing cells as controls. As expected, siRNA‐mediated CSE knockdown decreased H_2_S and CSE levels by approximately 60% and 65%, respectively (Figure 2C), whereas CSE‐Myc overexpression increased H_2_S and CSE levels by approximately 2.3‐fold and 3.0‐fold, respectively (Figure 2D), supporting H_2_S measurement specificity. These data suggested that the antihypertensive and vasorelaxatory activities of HNK are associated with its effect on H_2_S production.

### 3.3 HNK increases CSE protein levels by enhancing its stability against proteasomal degradation

**FIGURE 3:**
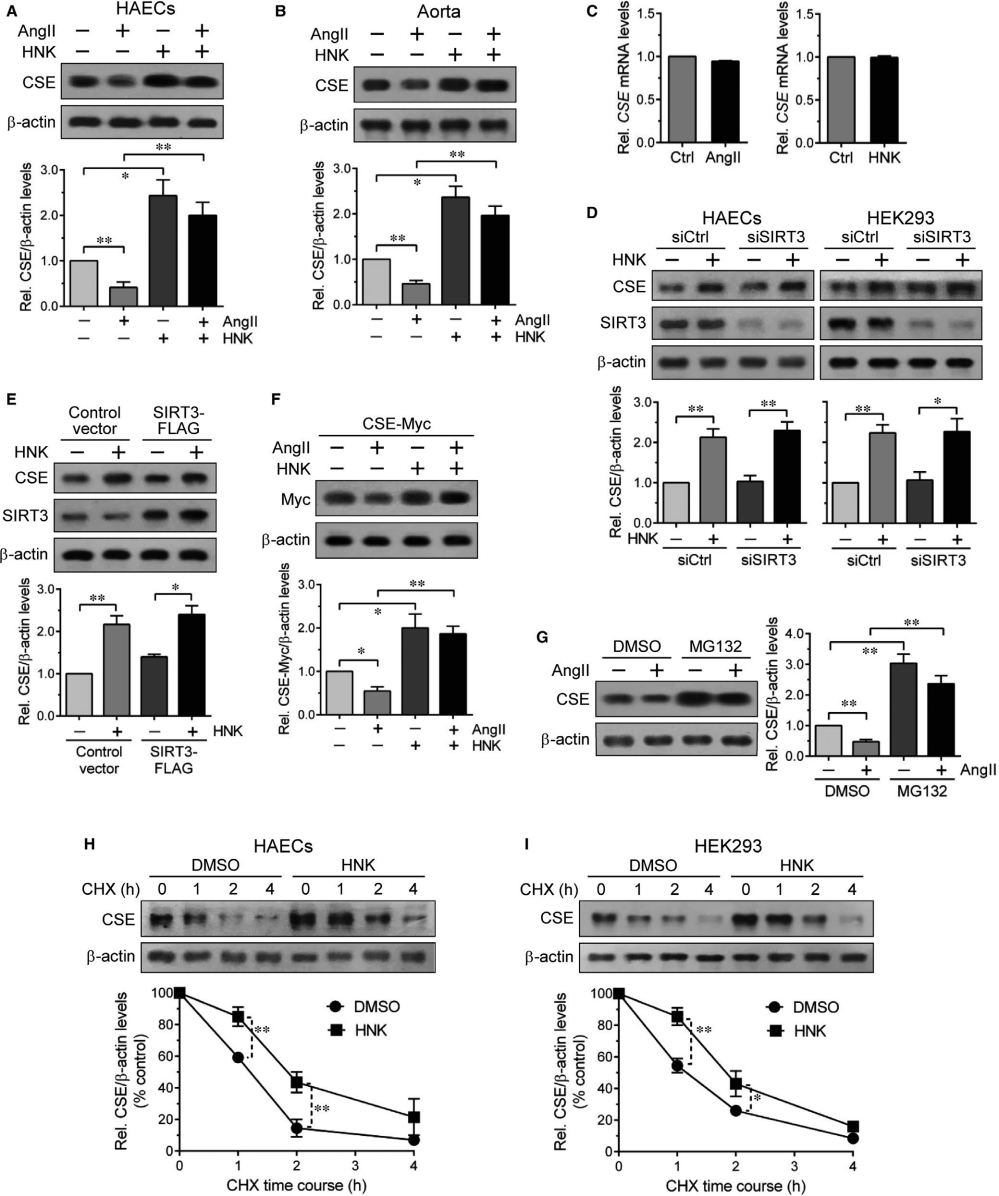
Reciprocal effects of AngII and HNK on CSE protein levels. A, HAECs pretreated with HNK (5 μmol/L) for 1 h were then left untreated or treated with AngII (100 nmol/L) for 12 h, as indicated. B, Aorta samples were isolated from the mice administered with AngII and/or HNK for 4 wk as described in Figure 1. CSE and β‐actin (a loading control) protein levels in HAEC (A) and aorta (B) lysates were detected by immunoblot analysis. C, *CSE* mRNA levels in HAECs treated with AngII or HNK were measured by qRT‐PCR analysis. CSE protein (A,B) and mRNA (C) levels were normalized to β‐actin and *GAPDH* levels, respectively, and quantified relative to those in the untreated control. Values represent the means ± SEM. *P < 0.05, **P < 0.01. D, HAECs or HEK293 cells were transfected with SIRT3 siRNA or control siRNA for 2 d. E, SIRT3‐FLAG or control vector plasmid was transfected into HEK293 cells for 1 d. F, HAECs transfected with CSE‐Myc plasmid for 1 d were processed with or without HNK (5 μmol/L, 1 h) pretreatment and/or AngII (100 nmol/L, 12 h) treatment. G, HAECs were pretreated with DMSO (as a control) or 10 μmol/L MG132 for 1 h, then incubated in the absence or presence of AngII (100 nmol/L) for 12 h. HAECs (H) or HEK293 cells (I) were incubated with 10 μmol/L CHX for the indicated times after pretreatment with DMSO or HNK (5 μmol/L) for 1 h. D‐I, The resulting cell lysates were immunoblotted with the indicated antibodies. Anti‐CSE (D,E,G‐I) and anti‐Myc (F) immunoreactivities were normalized to those of β‐actin and quantified relative to the untreated control (D‐G) or the respective zero time control (H,I). Values represent the means ± SEM. *P < 0.05, **P < 0.01

We next examined the effects of AngII and HNK on CSE protein levels in HAECs. AngII treatment decreased CSE protein levels and whereas HNK abrogated this down‐regulating effect, resulting in increased CSE protein levels (Figure 3A). We also tested mouse aorta samples that were prepared 4 weeks after AngII and/or HNK administration as described in Figure 1 and observed similar changes in CSE protein levels (Figure 3B). However, AngII and HNK minimally affected *CSE* mRNA levels, indicating that the CSE protein changes were not related to its transcription (Figure 3C). Considering that HNK is a potent activator of SIRT3,^26^ we examined whether SIRT3 is involved in HNK‐mediated CSE regulation using cells subjected to SIRT3 knockdown by siRNA. The control and SIRT3 knockdown HAECs and HEK293 cells showed almost equal increases in CSE protein levels upon HNK treatment (Figure 3D). Overexpression of SIRT3‐FLAG in HEK293 cells did not markedly alter HNK‐mediated CSE up‐regulation (Figure 3E). SIRT3 knockdown and overexpression efficiencies were confirmed by SIRT3 immunoblotting. These results indicated that HNK effects on CSE up‐regulation are not mediated through SIRT3.

Both endogenous CSE (Figure 3A) and transiently expressed CSE‐Myc levels in HAECs were oppositely regulated by AngII and HNK (Figure 3F). As the transfected CSE‐Myc is constitutively expressed by the cytomegalovirus promoter activity independently of endogenous gene expression, these results further suggested that HNK regulates CSE at the protein level. HAEC pretreatment with the proteasome inhibitor MG132 blocked AngII‐mediated CSE down‐regulation and MG132 alone also up‐regulated CSE protein levels (Figure 3G). We thus tested HNK for its effect on CSE protein stability using standard CHX chase experiments. Following HAEC treatment with CHX, an inhibitor of protein synthesis, CSE protein levels rapidly declined in control (DMSO only) conditions but remained relatively high in the presence of HNK during the experiment (Figure 3H). CHX chase assay in HNK‐treated HEK293 cells also showed a delay in time‐dependent CSE degradation (Figure 3I). These results indicated that CSE constitutes a protein substrate for proteasomal degradation under AngII‐stimulated and steady state conditions, which can be weakened by HNK.

### 3.4 HNK‐mediated HDAC6 inhibition induces CSE acetylation, which contributes to CSE up‐regulation

**FIGURE 4:**
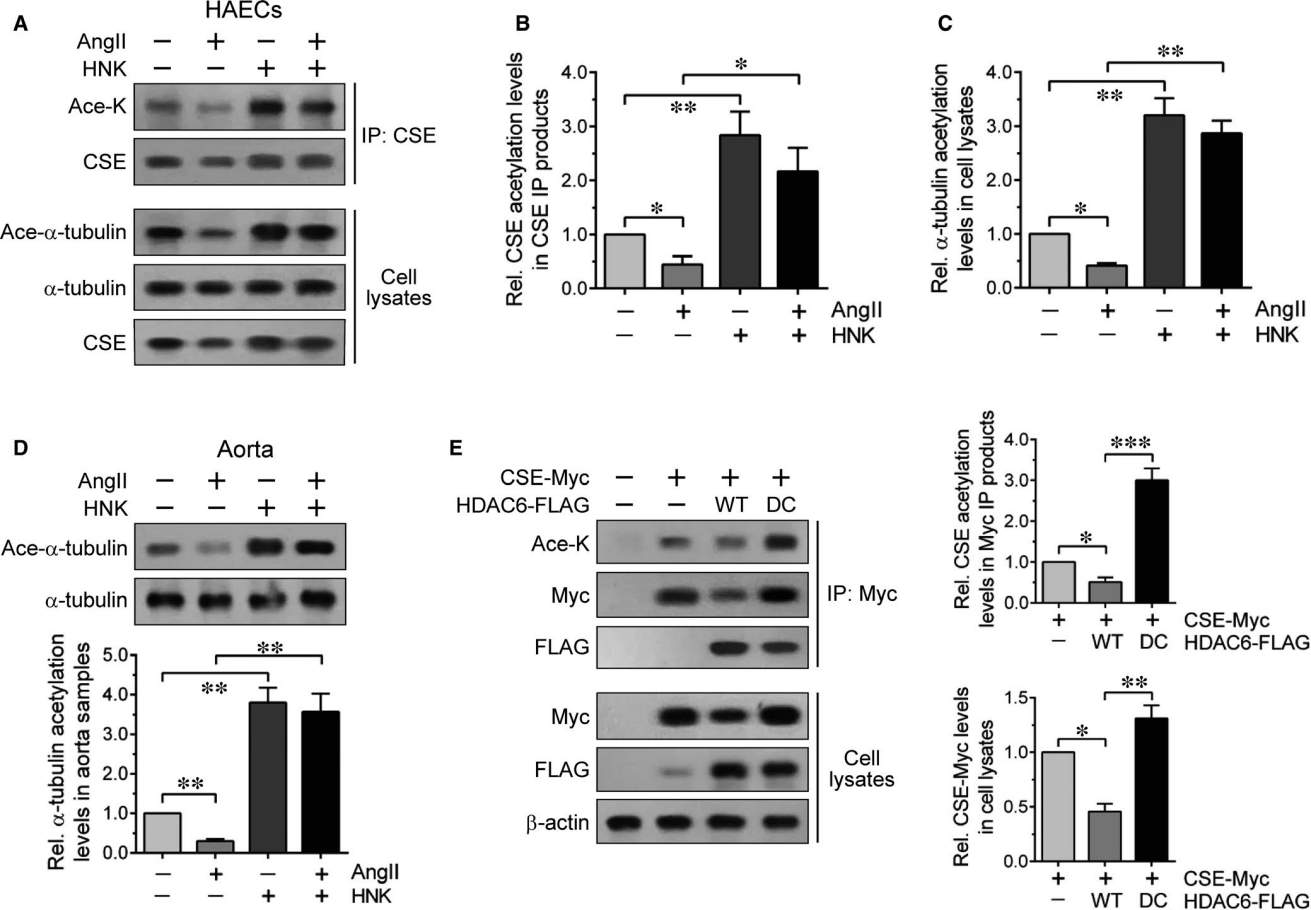
Increased acetylation levels of CSE and α‐tubulin by HNK. A, HAECs were treated with AngII (100 nmol/L) for 12 h following pretreatment with HNK (5 μmol/L) for 1 h, as indicated. Cell lysates were immunoprecipitated with anti‐CSE antibody, and then, the IP products and starting lysates were analysed by immunoblottings with the indicated antibodies including the anti‐Ace‐K and anti‐Ace‐α‐tubulin antibodies. (B,C) The ratios of Ace‐K and CSE immunoreactivities in the CSE IP products in (A) and Ace‐α‐tubulin immunoreactivities normalized to α‐tubulin immunoreactivities in cell lysates in (A), respectively, were quantified relative to the untreated control. Values represent the means ± SEM. *P < 0.05, **P < 0.01. D, Aorta samples were isolated from the AngII‐ and/or HNK‐infused mice as shown in Figure 3B. Ace‐α‐tubulin immunoreactivities were quantified as in (C). Values represent the means ± SEM. **P < 0.01. E, HEK293 cells cotransfected with wild‐type (WT) or inactive (DC) HDAC6‐FLAG together with CSE‐Myc, as indicated, were subjected to anti‐Myc IP. The cell lysates and IP products were analysed by immunoblotting with the indicated antibodies. The Ace‐K and Myc immunoreactivities in the Myc IP products and cell lysates, respectively, were quantified relative to those in the CSE‐Myc only. Values represent the means ± SEM. *P < 0.05, **P < 0.01, ***P < 0.001

As HDAC6‐mediated deacetylation of CSE promotes its proteasomal degradation,^20^ we examined whether HNK might modulate CSE acetylation levels through HDAC6. We thus analysed the CSE IP products prepared from AngII‐ and/or HNK‐treated HAEC by immunoblotting using an Ace‐K antibody to detect acetylated lysine residues. Immunoblot and quantification results revealed that whereas AngII reduced CSE acetylation levels, they were significantly increased by HNK regardless of AngII presence (Figure 4A,B). We assessed acetylation levels of α‐tubulin, an HDAC6 substrate, using an acetylation‐specific (K40) α‐tubulin (Ace‐α‐tubulin) antibody.^21^ Similar to the changes in CSE acetylation, the α‐tubulin acetylation levels were reciprocally regulated by AngII and HNK (Figure 4A,C). Additionally, AngII‐ and HNK‐treated mouse aorta samples also showed a noticeable decrease and increase in α‐tubulin acetylation levels, respectively (Figure 4D). Transfected CSE‐Myc acetylation levels were much higher in cells overexpressing a deacetylase‐deficient HDAC6 mutant than in cells overexpressing its wild‐type HDAC6 (Figure 4E). Moreover, the presence of coprecipitated HDAC6‐FLAG in the Myc IP product suggested a possible interaction between CSE and HDAC6. These results supported that HNK suppresses AngII‐induced CSE and α‐tubulin deacetylation mediated by HDAC6, leading to increased acetylation levels.

**FIGURE 5:**
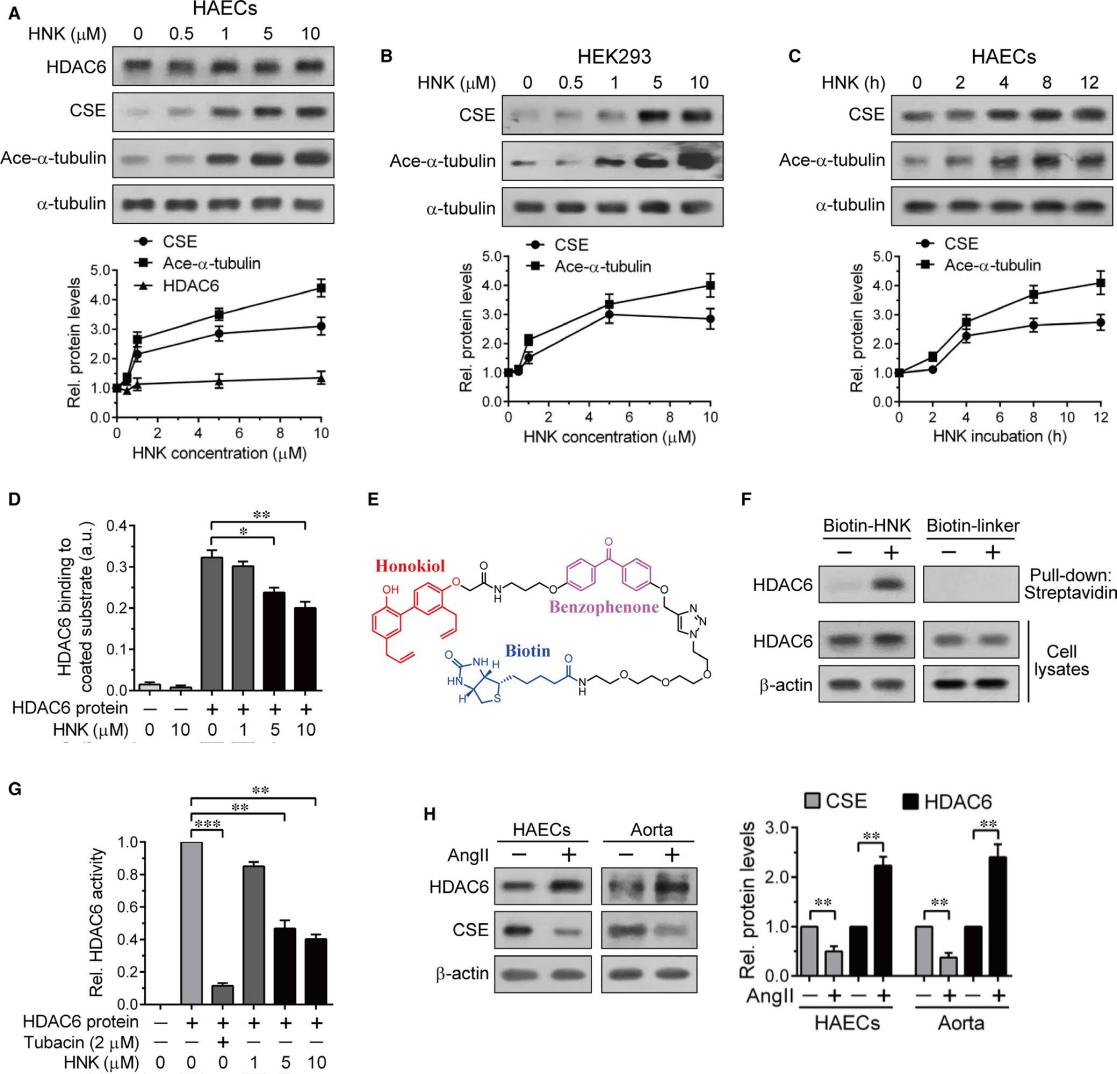
Inhibition of HDAC6 activity by HNK leading to CSE up‐regulation. HAECs (A) or HEK293 cells (B) were treated with the indicated concentrations of HNK for 12 h. C, HAECs were treated with 5 μmol/L HNK for the indicated times. (A‐C) The resulting cell lysates were immunoblotted with the indicated antibodies. The immunoreactivities of CSE, Ace‐α‐tubulin and/or HDAC6 were normalized to those of α‐tubulin and quantified relative to the untreated or zero time condition. Values represent the means ± SEM. D, Effects of HNK on HDAC6 binding to its unique HDAC6 affinity substrate were monitored using an ELISA‐based EpiQuik HDAC6 Assay Kit as described in Materials and Methods. After 2‐h incubation of purified HDAC6 protein in the absence or presence of the indicated concentrations of HNK in vitro, colorimetric changes were detected by measuring absorbance at 650 nm. Values represent the means ± SEM. *P < 0.05, **P < 0.01. E, The chemical structure of synthetic biotin‐labelled HNK (molecular weight: 1060.28) having a benzophenone‐containing linker. F, HAEC lysates were prepared 12 h after treatment with or without the biotin‐labelled HNK (5 μmol/L) or the biotin liker alone as a control and then pulled down with streptavidin agarose resins. The starting cell lysates and resulting pull‐down samples were subjected to immunoblot analysis with the indicated antibodies. G, In vitro HDAC6 activity was detected in the absence and presence of 2 μmol/L tubacin or the indicated concentrations of HNK by using a fluorometric HDAC6 activity assay kit as described in Materials and Methods. HDAC6 activity was quantified relative to the HDAC6 protein only condition. Values represent the means ± SEM. **P < 0.01, ***P < 0.001. H, Cell lysates from AngII‐treated HAECs and aorta extracts from AngII‐infused mice were prepared as shown in Figure 4A,D, respectively. Immunoreactivities of HDAC6 and CSE normalized to those of β‐actin were quantified relative to the untreated control. Values represent the means ± SEM. **P < 0.01

HNK up‐regulated α‐tubulin acetylation levels dose‐dependently in HAECs (Figure 5A) and HEK293 cells (Figure 5B). CSE protein levels showed the same dose‐dependent responses to HNK under the same conditions (Figure 5A,B). Similarly, both CSE protein and acetylated α‐tubulin levels were increased during the time course of HNK treatment in HAECs (Figure 5C). The positive correlation between CSE protein levels and α‐tubulin acetylation levels suggested that HNK contributes to CSE protein up‐regulation through inhibition of HDAC6 catalytic activity. Thus, we evaluated HNK binding to HDAC6 using a colorimetric, ELISA‐based EpiQuik HDAC6 Assay Kit that includes an unspecified affinity substrate‐coated plate and purified HDAC6 protein as a control. Although this assay kit is designed for measuring cellular HDAC6 protein levels, we merely performed in vitro incubation of the HDAC6 control protein with or without HNK. The HDAC6 ELISA revealed that the HDAC6 binding activities were dose‐dependently reduced by the presence of HNK (Figure 5D), suggesting that HNK–HDAC6 binding interferes with the HDAC6 binding affinity for the substrate.

Alternatively, we performed a pull‐down assay using a biotin‐labelled HNK (Figure 5E) recently synthesized for biological applications.^30^ Lysates from HAECs untreated or treated with biotin‐labelled HNK were pulled down using streptavidin agarose resins. The HDAC6 immunoblot analysis confirmed the binding of biotin‐labelled HNK to HDAC6 (Figure 5F). The streptavidin pull‐down assay with biotin‐linker alone (without HNK) showed no significant HDAC6 binding affinity (Figure 5F), supporting a specific binding between HNK and HDAC6. We also performed in vitro measurement of HDAC6 enzymatic activity using a fluorometric HDAC6 activity assay kit. Similar to an HDAC6 inhibitor tubacin, HNK suppressed HDAC6‐mediated deacetylation reaction at 5 and 10 μmol/L concentrations (Figure 5G), further supporting a direct inhibitory effect of HNK on HDAC6. When HNK was cotreated with tubastatin A, HNK exhibited an additive effect on HDAC6 inhibition, as shown by the relatively high α‐tubulin acetylation levels compared to those in tubastatin A only especially at a relatively low concentration (1 μmol/L) of tubastatin A (Figure S2). HDAC6 protein levels were not substantially affected by HNK (Figure 5A). In contrast, increased HDAC6 protein levels were accompanied with decreased CSE protein levels in AngII‐treated HAECs and mouse aortas (Figure 5H), which was consistent with the previous results.^20^ These data indicated that AngII‐mediated HDAC6 up‐regulation drives CSE deacetylation resulting in its degradation, whereas these changes are blocked by HNK owing to its inhibitory effect on HDAC6 activity.

### 3.5 CSE acetylation at K73 prevents its degradative ubiquitination

**FIGURE 6:**
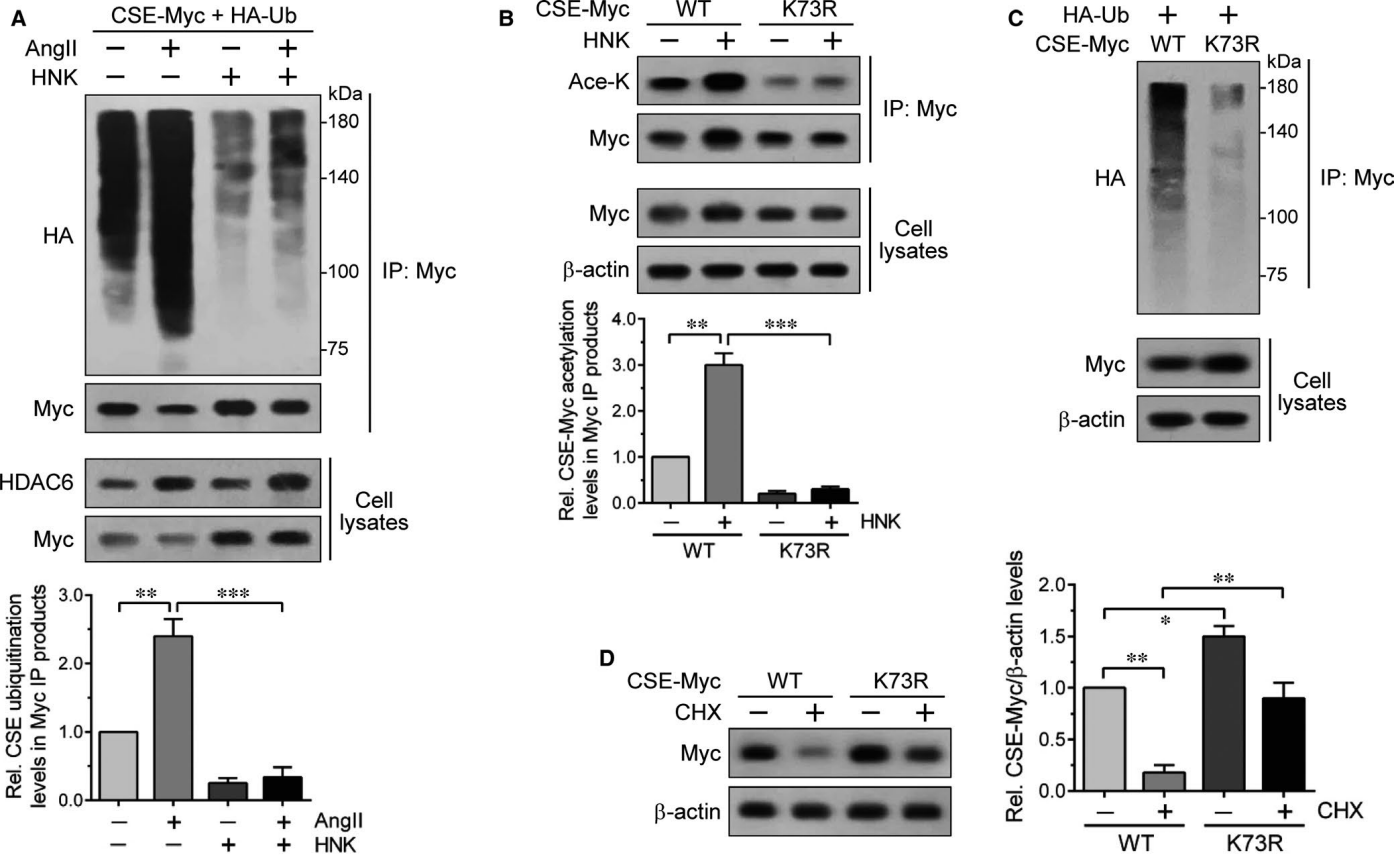
Regulation of CSE degradation by acetylation and ubiquitination of CSE at the K73 residue. A, HEK293 cells were cotransfected with CSE‐Myc and HA‐ubiquitin (Ub). At 1‐day post‐transfection, cells were incubated in the absence or presence of AngII and/or HNK as described in Figure 3F. B, HAECs transfected with CSE‐Myc wild‐type (WT) or K73R mutant were left untreated or treated with 5 μmol/L HNK for 8 h. C, HAECs were cotransfected with CSE‐Myc WT or K73R mutant together with HA‐Ub. A‐C, The resulting lysates were processed for anti‐Myc IP and the IP products and lysates were subjected to immunoblot analysis with the indicated antibodies. The HA (A) and Ace‐K (B) immunoreactivities in the Myc IP products were quantified relative to those in the untreated control and CSE‐Myc WT, respectively. Values represent the means ± SEM. **P < 0.01, ***P < 0.001. D, HEK293 cells transfected with CSE‐Myc WT or K73R mutant were incubated with or without 10 μmol/L CHX for 2 h. Cell lysates were immunoblotted with the indicated antibodies. The normalized Myc immunoreactivities were quantified relative to those in the untreated CSE‐Myc WT. Values represent the means ± SEM. *P < 0.05, **P < 0.01

Based on the AngII‐induced proteasomal degradation of CSE and the opposite regulation of CSE by AngII and HNK, we evaluated their potential effects on CSE ubiquitination using HEK293 cells cotransfected with CSE‐Myc and HA‐ubiquitin. As shown by the HA and Myc immunoblots of the anti‐Myc IP products, increased polyubiquitination of CSE‐Myc indicated by the multiple bands of higher molecular weight than wild‐type CSE‐Myc (approximately 45 kD) and its decreased levels, respectively, were clearly detectable under AngII treatment (Figure 6A), supporting CSE degradation via the ubiquitin‐proteasome system (UPS). Conversely, HNK strongly reduced CSE‐Myc polyubiquitination levels regardless of AngII (Figure 6A). As HNK increased CSE stability and its acetylation levels, these findings further suggested that the HNK‐induced CSE acetylation acts to inhibit the UPS‐mediated CSE degradation.

A previous proteomics study to identify ubiquitinated and acetylated protein substrates and their target lysine residues revealed that CSE undergoes both modifications and the K73 in CSE is a main target site.^36^ We thus generated a CSE‐Myc plasmid point mutant (K73R) and tested its acetylation and ubiquitination efficiency using transfection and anti‐Myc IP as described in Figure 4E and Figure 6A, respectively. As shown by the Ace‐K immunoblot of the Myc IP samples, K73R mutant acetylation levels were lower than those of its wild‐type CSE irrespective of HNK (Figure 6B). The HA immunoblot of the Myc IP samples also showed relatively low CSE ubiquitination levels in K73R‐expressing cells compared with those in wild‐type‐expressing cells (Figure 6C). As these results indicated that K73 is a major site of both CSE acetylation and ubiquitination, we hypothesized that CSE K73R becomes more stable than the wild‐type as the mutant is resistant to both acetylation and ubiquitination. To test this, we treated wild‐type‐ or K73R‐transfected cells with CHX. K73R protein levels were relatively higher than wild‐type, as expected (Figure 6D). The K73R protein levels in resting conditions (without CHX) also remained at high levels compared to the wild‐type (Figure 6D). Overall, these observations suggested that CSE is degraded through K73 ubiquitination, which can be facilitated by K73 deacetylation but prevented by its acetylation.

## 4 DISCUSSION

**FIGURE 7:**
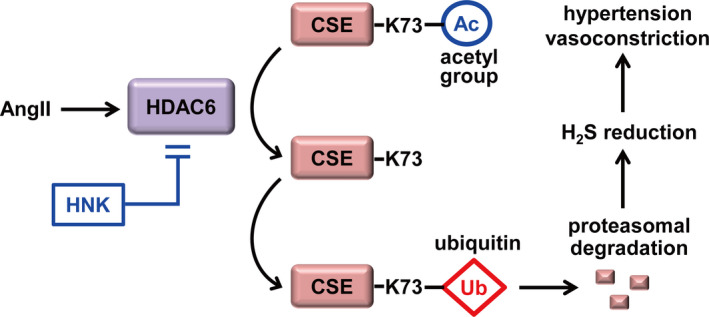
Working model for HNK‐mediated attenuation of AngII‐induced hypertension and endothelial dysfunction through HDAC6 and CSE. H_2_S produced by CSE stimulates endothelium‐dependent vasorelaxation, relieving hypertension. CSE undergoes proteasomal degradation through K73 ubiquitination, which is negatively regulated by its acetylation. AngII‐induced HDAC6 up‐regulation results in CSE deacetylation at K73, thereby promoting its degradative ubiquitination. HNK can act as an HDAC6 inhibitor. Accordingly, HNK enhances CSE acetylation levels and its protein stability. As a result, the enhanced H_2_S production contributes to prevent hypertension and endothelial dysfunction

In this study, we demonstrated that HNK contributes to ameliorate AngII‐induced hypertension and endothelial dysfunction by regulating the HDAC6‐mediated CSE degradation that is dependent on CSE K73 acetylation and ubiquitination (Figure 7). Our results showed that HNK reduces the AngII‐mediated blood pressure elevation and improves AngII‐attenuated endothelium‐dependent vascular relaxation. We also observed contrasting effects of HNK and AngII on CSE protein levels. HNK could enhance CSE protein stability whereas AngII promoted its degradation. Similarly, we showed that HNK counterbalanced AngII‐down‐regulated H_2_S levels. As CSE‐mediated H_2_S production induces vasodilation, consequently leading to the attenuation of hypertension,^4^, ^7^ our results support that the up‐regulation of CSE and H_2_S levels by HNK constitutes a primary mechanism underlying its antihypertensive and vasodilatory activities. It is known that H_2_S, the product of CSE, can crosstalk with eNOS, leading to enhanced NO production.^4^, ^32^, ^33^ As our results showed an eNOS activation by HNK, we cannot exclude the possibility that the HNK‐induced CSE up‐regulation can, at least partially, contribute to vasodilation through NO production.

We identified CSE as an acetylated and ubiquitinated protein substrate. Notably, our current results demonstrated that CSE acetylation status is coupled to its protein stability and degradation and that HDAC6 is critically engaged in HNK and AngII‐mediated CSE regulation. Mechanistically, AngII augmented HDAC6 expression to induce CSE deacetylation; the deacetylated CSE form, in turn, underwent proteasomal ubiquitination and degradation. Conversely, HNK could block the HDAC6‐dependent CSE deacetylation, which protected CSE from UPS‐mediated degradation. As HNK strongly increased CSE acetylation levels and, conversely, decreased its ubiquitination levels, it is likely that CSE acetylation plays a suppressive role in its degradative ubiquitination in a competitive manner. Overall, our results indicated that the reciprocal changes in CSE acetylation levels by AngII and HNK do not simply occur proportional to CSE protein levels but rather constitute a determining factor for CSE stability.

Protein acetylation and ubiquitination are common post‐translational modifications that target lysine residues. Our results further supported that the K73 of CSE is a major site for these modifications that is associated with the control of HDAC6‐dependent CSE ubiquitination. Previous studies have reported that HDAC6‐mediated deacetylation of target protein substrates triggers their degradation through the UPS pathway.^37^, ^38^ For example, heat‐shock protein 5 deacetylation by HDAC6 promotes its ubiquitination for proteasomal degradation.^39^ With regard to tumor suppressor MST1, a component of the Hippo pathway, HDAC6‐mediated deacetylation induced its degradation through chaperone‐mediated autophagy.^40^ The generation of reactive oxygen species (ROS) by AngII is closely associated with AngII‐induced various cardiovascular dysfunctions.^41^ HDAC6 has been shown to participate in pathological progressions involving oxidative stress. For example, HDAC6‐mediated deacetylation of peroxiredoxin 1 decreased its antioxidant activity, resulting in increased susceptibility to oxidative stress‐induced injury.^42^, ^43^ Application of HDAC6 inhibitors such as tubastatin A exerts a cytoprotective effect by reducing generation of ROS.^42^ In this context, our results indicating an enhanced HDAC6 protein expression by AngII further suggested that an increase in ROS plays a role in endothelial dysfunction and hypertension. In addition, a recent study reported that AngII induced UPS‐dependent CSE degradation and H_2_S reduction in vascular endothelial cells, which was similar to albeit somewhat different from our findings as AngII‐generated ROS mediated the CSE degradation.^44^ Considering the anti‐oxidative effects of HNK,^24^, ^45^ we cannot completely exclude the possibility that HNK may also negatively regulate the degradative ubiquitination of CSE by reducing ROS levels.

HDAC6 is a major α‐tubulin deacetylase; thus, changes in α‐tubulin acetylation status have been widely used for HDAC6‐related applications in vitro and in vivo.^21^, ^46^, ^47^, ^48^ Here, we provided evidence demonstrating the robust increases in α‐tubulin acetylation by HNK. We further ascertained the marked binding affinity of HNK for HDAC6 and the reducing effect of HNK on HDAC6 deacetylase activity. These suggested that HNK acts as an inhibitor of HDAC6. This result was consistent with the previous finding that HNK also increased the acetylation levels of heat‐ shock protein 90, another specific HDAC6 substrate.^49^ The reduction in α‐tubulin acetylation by HDAC6 is associated with microtubule destabilization.^47^ HNK was shown to alleviate cytotoxic damages of renal epithelial cells by promoting actin and tubulin polymerization and tubulin bundle formation.^50^ This suggested that HNK may contribute to enhanced cytoskeletal integrity by inhibiting HDAC6. Moreover, our current evidence is reminiscent of our previous results obtained using tubastatin A, the well‐established HDAC6 inhibitor.^51^ Tubastatin A also counteracted AngII hypertensive activity and blocked AngII‐induced CSE deacetylation and UPS‐dependent degradation.^20^ However, the precise mechanism by which HNK mediates HDAC6 inactivation remains to be elucidated.

Several lines of evidence have shown that HNK exerts a protective effect against various cardiovascular dysfunctions and that HNK‐mediated SIRT3 activation contributes to its cardioprotective activities.^26^, ^27^ SIRT3, an NAD^+^‐dependent protein deacetylase, has emerged as a critical regulator of metabolic processes in the mitochondria that contributes to maintaining cardiac function.^52^, ^53^ Conversely, the zinc‐dependent deacetylase HDAC6 plays a pathologic role in heart and vessel dysfunctions such as hypertension, atherosclerosis, cardiac hypertrophy and fibrosis.^13^, ^14^, ^15^, ^16^, ^17^, ^18^ HDAC6 is structurally unrelated to and different from SIRT3 in terms of subcellular localization, activation mechanism and target deacetylation substrates.^46^, ^54^, ^55^ Considering the differential aspects of HDAC6 and SIRT3, it might be expected that HNK ultimately leads to improvement of endothelial functions through HDAC6 inhibition in addition to SIRT3 activation. Here, our data showed that the HNK‐induced CSE up‐regulation was largely unaffected by SIRT3 knockdown or overexpression, supporting that the regulatory effects of HNK on CSE were SIRT3‐independent.

Overall, our current results suggested that HNK contributes to restoring various physiological impairments, at least in part, by impeding the HDAC6‐mediated CSE degradation. In conclusion, we demonstrated for the first time that HNK‐mediated HDAC6 inhibition induces acetylation‐dependent CSE stabilization, thereby attenuating AngII‐induced hypertension and endothelial dysfunction.

## CONFLICT OF INTEREST

The authors declare no conflict of interest.

## AUTHOR CONTRIBUTION

**Zhexi Chi:** Conceptualization (supporting); Data curation (lead); Formal analysis (lead); Investigation (lead); Methodology (lead); Project administration (supporting); Validation (lead); Visualization (lead). **Truc Phan Hoang Le:** Formal analysis (lead); Investigation (lead); Validation (supporting). **Sang Ki Lee:** Formal analysis (supporting); Investigation (supporting); Software (supporting); Validation (supporting). **Erling Guo:** Formal analysis (supporting); Investigation (supporting); Software (supporting); Validation (supporting). **Dongsoo Kim:** Formal analysis (supporting); Investigation (supporting); Validation (supporting). **Sanha Lee:** Formal analysis (supporting); Investigation (supporting); Resources (supporting); Validation (supporting). **Seung‐Yong Seo:** Data curation (supporting); Resources (supporting). **Sook Young Lee:** Data curation (supporting). **Jae Hyung Kim:** Conceptualization (lead); Data curation (supporting); Funding acquisition (lead); Methodology (supporting); Project administration (supporting); Supervision (lead); Writing‐original draft (lead). **Sang Yoon Lee:** Conceptualization (lead); Data curation (lead); Funding acquisition (lead); Methodology (lead); Project administration (lead); Supervision (lead); Visualization (lead); Writing‐original draft (lead); Writing‐review & editing (lead). 

## Supporting information

Fig S1‐S2

## Data Availability

This manuscript does not contain sharable data.
