# Supplementary material for: Honokiol ameliorates angiotensin II‐induced hypertension and endothelial dysfunction by inhibiting HDAC6‐mediated cystathionine γ‐lyase degradation
Source: J Cell Mol Med. 2020 Aug 4;24(18):10663–76. doi: 10.1111/jcmm.15686 (PMC7521302; doi:10.1111/jcmm.15686)
Supplement: Supplementary file 1 — Fig S1‐S2 [file JCMM-24-10663-s001.docx]

**Supplementary figures**

**
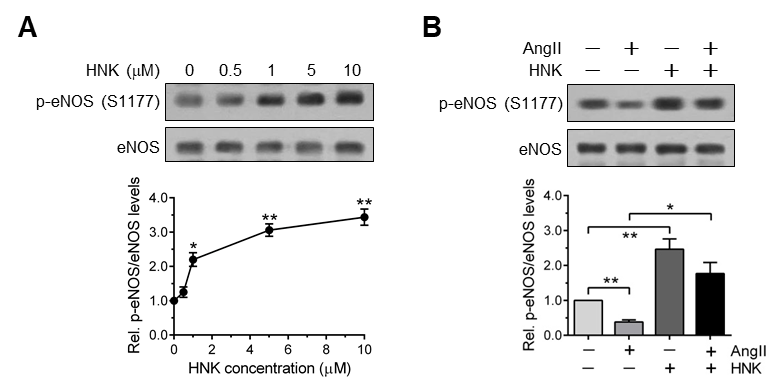
**

**FIGURE S1** Increased eNOS phosphorylation (S1177) by HNK. (A) HAECs were treated with the indicated concentrations of HNK for 12 h. (B) HAECs were pretreated with HNK (5 μM) for 1 h and then treated with AngII (100 nM) for 12 h, as indicated conditions. Resulting cell lysates were analyzed by immunoblottings with the phospho-eNOS (S1177) and eNOS antibodies. The immunoreactivities of phospho-eNOS were normalized to those of eNOS and quantified relative to the untreated control. Values represent the means ± SEM. **p* < 0.05, ***p* < 0.01.

**
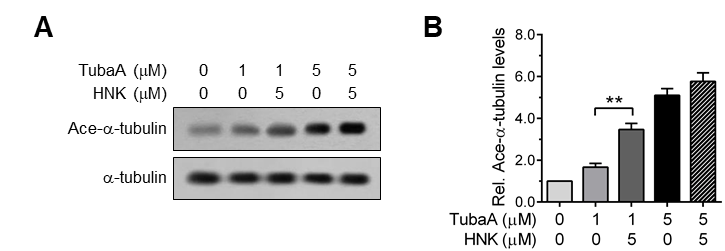
**

**FIGURE S2** A combined effect of HNK and tubastatin A (TubaA) on α-tubulin acetylation. (A) HAECs were treated without or with HNK in the presence of TubaA for 12 h, as indicated conditions. Cell lysates were immunoblotted with the indicated antibodies. (B) Acetylated α-tubulin immunoreactivities normalized to α-tubulin immunoreactivities in (A) were quantified relative to the untreated control. Values represent the means ± SEM. ***p* < 0.01.
